# Web-Based Information on Spinal Cord Stimulation: Qualitative Assessment of Publicly Accessible Online Resources

**DOI:** 10.2196/50031

**Published:** 2024-02-23

**Authors:** Tiev Miller, Ali Hosseinzadeh, Thomas Thordarson, Tamila Kalimullina, Soshi Samejima, Claire Shackleton, Raza Malik, Martín Calderón-Juárez, Rahul Sachdeva, Andrei Krassioukov

**Affiliations:** 1 International Collaboration on Repair Discoveries The University of British Columbia Vancouver, BC Canada; 2 Division of Physical Medicine and Rehabilitation Department of Medicine The University of British Columbia Vancouver, BC Canada; 3 Department of Rehabilitation Medicine University of Washington Seattle, WA United States; 4 Spinal Cord Program GF Strong Rehabilitation Centre Vancouver Coastal Health Vancouver, BC Canada

**Keywords:** access to information, consumer health information, internet, spinal cord stimulation, web-based information, communication, quality, readability, Google Trends, misinformation, synthesis

## Abstract

**Background:**

Despite the growing accessibility of web-based information related to spinal cord stimulation (SCS), the content and quality of commonly encountered websites remain unknown.

**Objective:**

This study aimed to assess the content and quality of web-based information on SCS.

**Methods:**

This qualitative study was prospectively registered in Open Science Framework. Google Trends was used to identify the top trending, SCS-related search queries from 2012 to 2022. Top queried terms were then entered into separate search engines. Information found on websites within the first 2 pages of results was extracted and assessed for quality using the DISCERN instrument, the Journal of the American Medical Association benchmark criteria, and the Health on the Net Foundation code of conduct certification. Website readability and SCS-related information were also assessed.

**Results:**

After exclusions, 42 unique sites were identified (scientific resources: n=6, nonprofit: n=12, for-profit: n=20, news or media: n=2, and personal or blog: n=2). Overall, information quality was moderate (DISCERN). Few sites met all the Journal of the American Medical Association benchmark criteria (n=3, 7%) or had Health on the Net Foundation certification (n=7, 16%). On average, information was difficult to read, requiring a 9th- to 10th-grade level of reading comprehension. Sites described SCS subcategories (n=14, 33%), indications (n=38, 90%), contraindications (n=14, 33%), side effects or risks (n=28, 66%), device considerations (n=25, 59%), follow-up (n=22, 52%), expected outcomes (n=31, 73%), provided authorship details (n=20, 47%), and publication dates (n=19, 45%). The proportion of for-profit sites reporting authorship information was comparatively less than other site types (n=3, 15%). Almost all sites focused on surgically implanted SCS (n=37, 88%). On average, nonprofit sites contained the greatest number of peer-reviewed reference citations (n=6, 50%). For-profit sites showed the highest proportion of physician or clinical referrals among site types (n=17, 85%) indicating implicit bias (ie, auto-referral).

**Conclusions:**

Overall, our findings suggest the public may be exposed to incomplete or dated information from unidentifiable sources that could put consumers and patient groups at risk.

## Introduction

### Background

Spinal cord stimulation (SCS) is an emerging therapeutic approach that has been used as an intervention to address chronic pain [1,2], paralysis, and autonomic dysfunctions [3,4] resulting from injury and disease. SCS delivers electrical stimulation to the spinal cord using invasive or noninvasive interfaces. Groundbreaking pilot studies involving individuals with paralysis after spinal cord injury have demonstrated the potential benefits of SCS for improving functional recovery [5,6], resulting in a growing demand for information related to SCS among various consumer or patient groups, their caregivers, and the general public [7]. In the absence of evidence-based guidelines and standardized treatment options, people living with long-term disease and disability are likely to turn to other sources of information (eg, web-based search results), and potentially alternative treatment options (eg, advertised devices and surgical procedures), to help manage their condition. However, web-based information on SCS has not been evaluated in depth.

Currently, the US Food and Drug Administration [8] has only approved the use of epidural spinal cord stimulation (ESCS; ie, surgically implanted) to manage failed back surgery syndrome, refractory angina pectoris, peripheral arterial disease, complex regional pain syndrome, painful diabetic neuropathy as well as nonsurgical low back pain. Whether publicly accessible websites curating information on SCS provide clear indications (or contraindications) for the intended use (or potential risk) of this therapeutic modality remains unknown. Over the next decade, medical consumerism is projected to grow as people seeking treatment become more knowledgeable and active in their care through the expansion of web-based resources and the availability of medical information [9]. As the marketing of SCS to treat or manage various conditions expands concomitantly with the fragmentation of web-based medical information [10] and the gradual erosion of routine patient-to–health care provider interactions [11], patients may become more vulnerable to predatory marketing strategies that inflate the benefits of SCS while obscuring or underreporting its potential harms [12]. This market-driven shift by which medical information is being provided and subsequently accessed may engender patient consumerism and potentially enhance the pecuniary opportunities for SCS device manufacturers and health care providers [13].

### Rationale

Many internet searches are health-related (eg, 4.5% of 6.75 million daily searches in 2003) and are usually conducted using the most popular search engines (eg, Google) [14,15] rather than by directly accessing individual websites exclusively dedicated to the provision of medical information. Though the amount of published research on the topic of SCS has risen exponentially in the past decade [7], the pervasiveness of health-related misinformation on publicly accessible web-based platforms has grown in tandem [13,16]. Previous studies have shown that misinformation is commonly encountered on sites describing disease prevalence [10,17], evaluation [18], available treatment options [19], and medical management recommendations [20-22].

Misinformation may present as information that is either lacking in critical detail, misleading or factually incorrect, or out-of-date and not substantiated by current evidence [16]. The amount, quality, and relevance of the information returned by a given search engine query may vary substantially [23]. Website order in a returned search is also important, as preferential positioning determines what information (eg, medical treatment and management) is accessed first, independent of content [24] and quality [25]. Most user traffic (ie, 92%) is often confined to the top results found within the first search page, with roughly one-third of all user traffic attributed to the first listed result [26]. Furthermore, the provision of health-related information on websites with substantial user traffic is often inconsistent with evidence-based guidelines and best practice recommendations [25,27]. As the pursuit and use of web-based health-related information continues to outpace and even remodel conventional patient-to-health care provider interactions [11,13], it is important that web-based information is critically appraised.

### Objectives

The objectives of this qualitative study were (1) to systematically map web-based resources containing SCS-related information using common search methods that are openly and freely available to the general public and (2) to assess the content and quality of information regarding the use of SCS for the treatment or management of health conditions or symptoms.

## Methods

### Ethical Considerations

Ethics approval was not required for this study, as it did not involve human participants.

### Search Strategy and Inclusion Criteria

**Figure 1:**
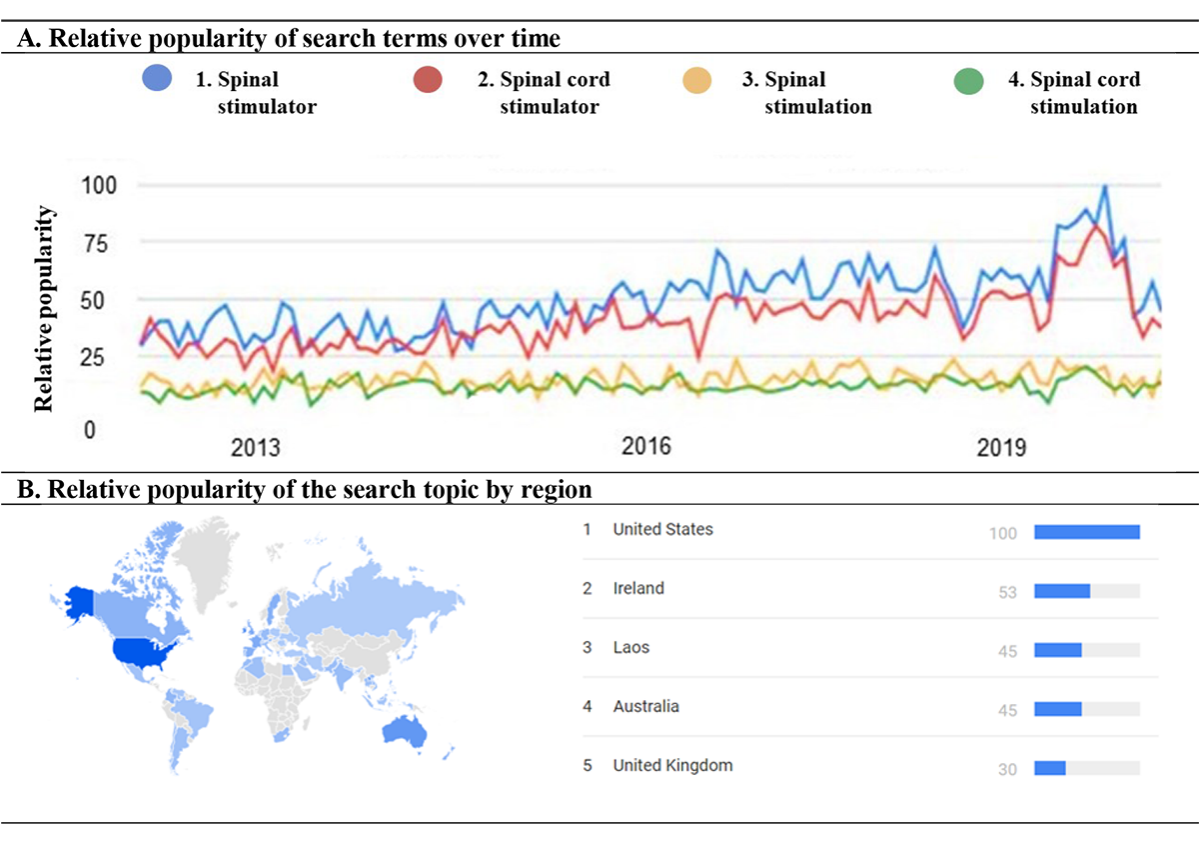
Search term identification. (A) Search terms with relevance to spinal cord stimulation were identified using Google Trends. Topic search using the term “spinal cord stimulator” was used to identify the top related queries: 1=spinal stimulator, 2=spinal cord stimulator, 3=spinal stimulation, and 4=spinal cord stimulation. Among the top queried terms, 1=spinal stimulator and 2=spinal cord stimulator ranked the highest in terms of relative search term popularity. (B) Search interest for the topic term “spinal cord stimulator” by global region indicated the United States (n=100), followed by Ireland (n=53), Laos (n=45), Australia (n=45), and United Kingdom (n=30) had the highest proportion of all queries (ie, not the highest number of queries) for this topic term within the specified timeframe (2012-2022).

This study was prospectively registered in Open Science Framework [28]. A pilot search was conducted to identify the most popular search terms (worldwide) within the last decade (2012 to 2022) for the topic “spinal cord stimulator” using Google Trends (Figure 1). All searches were performed using a United States IP designation to standardize the country-specific origin of returned results for each search engine [17]. A detailed description of the search strategy using the highest trending search queries (ie, “spinal stimulator” and “spinal cord stimulator”) is provided in the *Search Strategy* section in Multimedia Appendix 1 [17,28-77].

This study considered publicly accessible websites providing information on SCS. Websites were categorized as scientific resources (ie, academic institutions and government organizations), nonprofit or foundations or advocacy organizations, for-profit or private sector or industry, news or media, or independent sources or personal blogs in accordance with subcategorizations previously outlined by Fisher et al [17]. Sites were not excluded on the basis of origin or date. Peer-reviewed papers or book chapters hosted by journal publisher websites were not included in the data extraction. Evidence suggests the general public may be unable to interpret or understand all scientific content found within peer-reviewed literature (ie, accessibility or readability of information) [78]. As this content has already been subjected to professional scrutiny from subject-area experts and editorial revision of considerable rigor or is altogether inaccessible (eg, paywall restricted), this information was not evaluated. Advertisement sites that did not include information relevant to SCS were also excluded.

### Data Extraction

Data were extracted by 2 independent authors, and discrepancies were resolved through discussion or consensus with a third author. Extracted data included website type [17], characteristics (ie, site title, address [URL], publication date or most recent update, author names and credentials, country of origin or geographical designation, alternate accessibility options (format), and number of peer-reviewed reference citations), and SCS-related information provided (ie, SCS definition or summary, indications, contraindications, side effects or risks, device considerations, follow-up, outcomes, intended audience [consumer or caregiver, researcher or clinician, or both], and referral to a health care professional [eg, physician and therapist] or clinic).

### Quality and Readability

In accordance with methods described in previous studies [17,18], the quality of the information provided on each website was assessed by 2 independent authors using the DISCERN instrument [29], the Journal of the American Medical Association (JAMA) benchmark criteria [30], and Health on the Net Foundation (HON) code of conduct certification [31]. Readability for each site was determined using the Flesch-Kincaid indices [32]. These assessments are described in detail in the *Quality and Readability Assessments* section in Multimedia Appendix 1.

### Data Synthesis

Data extracted from websites were tabulated and aggregated according to site type. Websites were also explored for high-frequency keywords. Webpages were imported as PDF to NVivo software (version 12; QRS International Pty Ltd) and archived for further analysis using the NCapture extension in Google Chrome. Website text from imported PDF files was then used to generate word clouds and frequency tables. Descriptive analyses were performed using SPSS software (version 28; IBM Corp). Average scores and ratings for each site type are expressed as mean and SD, median and IQR, or frequencies (n) and proportions (%) for continuous, ordinal, and dichotomous levels of data, respectively.

## Results

### Screening and Search Term Frequency

Using the term “spinal stimulator” in the first search and “spinal cord stimulator” in the second search (Figure 1), a total of 202 results were returned (Google: n=56, Baidu: n=39, Yahoo: n=58, and Bing: n=49). After exclusions (ie, duplicates, advertisements, and sites unrelated to SCS), a total of 57 unique websites were identified. Of these, 15 were host sites for peer-reviewed journal papers (n=8), book chapters (n=4), or patent applications (n=3) and were thus excluded from the main synthesis. Of the remaining 42 sites, the majority were categorized as for-profit (n=20) followed by nonprofit (n=12), scientific resource (n=6), news or media (n=2), and personal or blog site types (n=2). A flow diagram outlining the screening procedure is provided in Table 1.

Word frequencies for each search term used (ie, spinal, cord, and stimulator) are provided in Table 2 and Figure S1 in Multimedia Appendix 1. Word cloud and word frequency summaries are also provided in Figure S1 in Multimedia Appendix 1. Word frequency summaries showed that the occurrence of individual words comprising the search topic (ie, “spinal,” “cord,” and “stimulator”) was among the most frequent. This suggests the returned search results are likely to be an accurate representation of the web-based resources and SCS-related information that consumers would encounter irrespective of the search engine used [79,80]. Average occurrence (ie, frequency) of all 3 search terms was highest for scientific resource sites (spinal: mean 25.7, SD 16; cord: mean 23.7, SD 14.5; and stimulator: mean 13.8, SD 13.3), while proportional usage (ie, relative to all other words) was highest among personal or blog sites for the terms “spinal” (mean 4.4, SD 2.7) and “stimulator” (mean 3.5, SD 3.2) and news or media sites for the term “cord” (mean 5.4, SD 6.8).

**Table 1:** Search results and website selection.

Screening procedure		Search engine			
		Google, n (%)	Baidu, n (%)	Yahoo, n (%)	Bing, n (%)
Initial search results (n=202)		56 (28)	39 (19)	58 (29)	49 (24)
Unique sites after duplicates (n=57)		23 (40)	15 (26)	6 (11)	13 (23)
**Eligibility criteria not met**					
	Journal papers (n=8)	3 (37)	5 (63)	—^a^	—
	Book chapters (n=4)	2 (50)	—	1 (25)	1 (25)
	Patent applications (n=3)	—	3 (100)	—	—
	Included websites (n=42)	18 (43)	7 (17)	5 (12)	12 (28)

^a^Not available.

**Table 2:** Quality, readability, and search term frequency.

Website category		Scientific resource (n=6)		Nonprofit (n=12)		For-profit (n=20)		News or media (n=2)		Personal or blog (n=2)
**DISCERN, median (IQR)**										
	Item 1 (clear aims)	5 (5-5)	5 (5-5)		5 (5-5)		5 (5-5)		5 (5-5)	
	Item 2 (achieved aims)	5 (5-5)	5 (5-5)		5 (5-5)		5 (5-5)		5 (5-5)	
	Item 3 (relevant)	5 (4.5-5)	5 (5-5)		5 (5-5)		5 (5-5)		5 (5-5)	
	Item 4 (information source)	1 (1-2)	1 (1-5)		1 (1-1)		3 (1-3)		1 (1-1)	
	Item 5 (publication date)	2 (1-5)	5 (1-5)		1 (1-1)		5 (5-5)		1 (1-1)	
	Item 6 (balanced and unbiased)	5 (3.25-5)	5 (1.5-5)		1.5 (1-3)		2.5 (2-2.5)		3 (2-3)	
	Item 7 (additional information sources)	2 (1-3.5)	3 (1-5)		1 (1-3)		3 (1-3)		2.5 (1-2.5)	
	Item 8 (areas of uncertainty)	3.5 (2.75-5)	3 (1-5)		3 (2-3.75)		4 (4-4)		2 (1-2)	
	Item 9 (defines treatment)	5 (4.75-5)	4 (1.25-5)		5 (4-5)		3 (1-3)		3 (1-3)	
	Item 10 (describes benefits)	5 (5-5)	5 (1.5-5)		5 (2-5)		5 (5-5)		5 (5-5)	
	Item 11 (describes risks)	5 (1-5)	4 (1-5)		2 (1-5)		3.5 (2-3.5)		1 (1-1)	
	Item 12 (no treatment or usual care outcomes)	1 (1-2)	1 (1-4)		1 (1-1.75)		1 (1-1)		3 (1-3)	
	Item 13 (effect on quality of life)	5 (4.5-5)	5 (1-5)		5 (2.25-5)		5 (5-5)		5 (5-5)	
	Item 14 (alternative treatment choices)	2 (1-5)	1.5 (1-5)		1 (1-5)		2 (1-2)		3 (1-3)	
	Item 15 (shared decision-making support)	3 (2.75-5)	3 (1-4)		3 (3-3.75)		3 (3-3)		3 (1-3)	
	Item 16 (overall)	3.5 (2.75-4)	3 (2.25-4)		3 (2-3)		2 (2-2)		2.5 (2-2.5)	
**JAMA^a^ benchmark criteria, median (IQR)**										
	Benchmark 1 (authorship)	1 (0.38-1)	1 (0.63-1)		0 (0-0.38)		1 (1-1)		1 (1-1)	
	Benchmark 2 (attribution)	0 (0-0.25)	0 (0-1)		0 (0-0)		0 (0-0)		0 (0-0)	
	Benchmark 3 (currency)	1 (0.75-1)	1 (1-1)		1 (1-1)		0.75 (0.5-0.75)		1 (1-1)	
	Benchmark 4 (disclosure)	0 (0-0.25)	0.5 (0-1)		0 (0-0.5)		0.25 (0-0.25)		0 (0-0)	
	Benchmark (total)	2 (0.88-2.5)	2.75 (2-3)		1.25 (1-1.9)		2 (1.5-2)		2 (2-2)	
HON^b^ code of conduct certificate, n (%)		2 (33)		4 (33)		1 (5)		0 (0)		0 (0)
**Readability, mean (SD)**										
	Flesch reading ease score	47.13 (11.54)	52.19 (7.84)		45.73 (10.66)		43.80 (16.69)		60.00 (19.94)	
	Flesch-Kincaid grade level	8.63 (1.72)	7.95 (1.18)		9.39 (1.88)		15.15 (9.26)		6.70 (3.54)	
**Search term word frequency, mean (SD)**										
	“Spinal” frequency	25.67 (16.02)	14.33 (13.39)		18.65 (19.22)		12.00 (15.56)		11.00 (12.73)	
	“Spinal” (%)	4.07 (2.18)	2.79 (2.07)		3.19 (1.89)		0.85 (0.47)		4.42 (2.71)	
	“Cord” frequency	23.67 (14.51)	13.92 (13.03)		17.40 (17.50)		10.50 (13.44)		9.00 (9.90)	
	“Cord” (%)	3.94 (2.26)	2.77 (2.00)		3.03 (1.79)		5.36 (6.84)		3.78 (1.81)	
	“Stimulator” frequency	13.83 (13.27)	4.83 (6.82)		11.95 (13.26)		9.00 (11.31)		9.50 (12.02)	
	“Stimulator” (%)	1.70 (1.48)	0.88 (1.37)		1.91 (1.43)		0.70 (0.25)		3.48 (3.15)	

^a^JAMA: Journal of the American Medical Association.

^b^HON: Health on the Net Foundation.

### Readability

A summary of readability is provided in Table 2 and Table S1 in Multimedia Appendix 1. Readability indices across all sites indicated that information was difficult on average and required a 9th- to 10th-grade level of reading comprehension (reading ease: mean 48.4, SD 10.8 and grade: mean 9, SD 2.7). News or media required the highest level of reading comprehension (mean 15.2, SD 9.3), and personal or blog sites required the lowest level of reading comprehension (mean 6.7, SD 3.5).

### Quality

A summary of information quality (ie, DISCERN, JAMA scores, and HON code of conduct certification) is provided in Table 2 and Figure S2 and Table S2 in Multimedia Appendix 1. Based on DISCERN rating criteria, just over one-third of all sites were rated as having high overall quality (n=16, 38%), with the remainder rated as having moderate (n=19, 45%) or low (n=7, 16%) overall quality. Scientific resources (median 3.5, IQR 2-3) and news or media sites (median 2, IQR 2-2) showed the highest and lowest overall DISCERN scores, respectively. Few sites met all 4 JAMA benchmark criteria (n=3, 7%). Nonprofit (median 2.75, IQR 2-3) and for-profit sites (median 1.25, IQR 1-1.9) showed the highest and lowest benchmark totals, respectively. HON code of conduct certification was present in less than a quarter of all sites (n=7, 16%), with the majority attributed to nonprofit sites (n=4, 9%).

### Website Characteristics

Tabulated website characteristics are summarized in Tables S3 and S4 in Multimedia Appendix 1. Publication dates (or latest revision date) were reported for less than half (19/42, 45%) of all websites and was proportionately lowest among for-profit sites (5/20, 25%) compared to other site types. Author names, aliases, and credentials were provided in less than half of all sites (20/42, 47%), and authorship disclosure was also proportionately lowest among for-profit sites (3/20, 15%) compared to others. Site addresses included designation in the United States (30/42, 71%), Canada (1/42, 2%), and Australia (1/42, 2%) or did not provide a location (10/42, 23%). Relative to other site types, the proportion of sites that provided a geographical designation was highest among for-profit sites (19/42, 45%). Just under half of all websites provided alternate content accessibility options (ie, PDF, audio, and video; 20/42, 47%). Some sites also featured pop-up notifications and advertisements (9/42, 21%). On average, nonprofit sites contained the greatest number of reference citations (mean 11.1, SD 27.1) followed by scientific resources (mean 1.2, SD 2.9).

### Website Information

Website information is summarized in Tables S3 and S5 in Multimedia Appendix 1. Most websites were intended for consumers and caregivers (n=27, 64%), with the remainder intended to serve as resources for clinicians and researchers (n=1, 2%) or both (n=14, 33%). Site information pertained to the use of ESCS (n=37, 88%), both transcutaneous SCS and ESCS (n=2, 4%), or did not define SCS type (n=3, 7%). Sites also provided summary information or a definition of SCS (n=37, 88%) and described SCS subcategories (n=14, 33%), indications (n=38, 90%), contraindications (n=14, 33%), side effects or risks (n=28, 66%), device considerations (n=25, 59%), follow-up (n=22, 52%), and outcome-related information (n=31, 73%). Most sites featured physician or clinical referral (n=26, 61%). Relative to other site types, for-profit sites showed the highest proportion of referrals (17/20, 85%).

## Discussion

### Principal Findings

This study provides a novel qualitative synthesis of publicly available web-based information on the topic of SCS. For-profit websites were the most commonly encountered. Website quality ratings varied between site types suggesting that the reporting (or omission) of important quality criteria (eg, authorship, information source, and publication date) was site-type dependent. For-profit sites were rated as having relatively lower quality than nonprofit or scientific resource sites based on quality criteria (ie, JAMA benchmarks and HON code of conduct certification). The proportion of for-profit sites reporting authorship was comparatively less than nonprofit and scientific resource sites. The average number of peer-reviewed reference citations identified within for-profit sites was also comparably less frequent than that of nonprofit and scientific resource sites. These findings are largely consistent with other studies of web-based information that report similar discrepancies in the provision of authorship and information sources [17,18]. This suggests that consumers may be exposed to web-based information on SCS that originates from unidentified authors and sources. While two-thirds of all websites mentioned potential risks or side effects of SCS, only one-third described possible contraindications for SCS. Moreover, the majority of websites specifically targeted consumers or their caregivers and featured clinical or physician referral for SCS. Relative to other site types, for-profit sites constituted the largest proportion of referrals indicating potential information bias and consumer-directed marketing of SCS [13]. This is a cause for concern, as the selective provision of SCS-related information from undisclosed sources may potentially place consumers and patient groups at unnecessary risk and consequently diminish their capacity to make informed health care decisions.

### Limitations

This study has several limitations. First, returned search results were limited by geographical location (ie, IP designation within the United States based on geographical trends for the search topic “spinal cord stimulator”) and may lack generalizability across regions and therefore introduce bias unintentionally. This study also incorporated the use of Baidu, the second-most commonly used search engine by international ranking at the time the search was conducted, in conjunction with other common search engines (ie, Google, Bing, and Yahoo). Although Baidu is the most commonly used web-based resource for medical information seekers following the withdrawal of Google from China in 2010 [81], the additive benefit of using this search engine and associated indices (eg, Baidu Index) to include a broader user demographic and potentially enhance search result generalizability remains inconclusive.

For-profit websites were the most commonly encountered site type, representing approximately half of all included sites (n=20, 48%). However, it is unclear whether the developers of these sites allocated funding and resources to increase user traffic and enhance website visibility through the use of strategic search engine optimization techniques (eg, content management systems; schematic data structuring; and technical optimizations to improve URL structure, loading, navigation, and internal linking). Although the results of the word frequency summary potentially suggest the use of word frequency–based ranking algorithms, the way these search engines ultimately select which resources to display in a given list of returned search results remains unknown. Future studies may investigate whether academic institutions and government and nongovernment organizations (ie, scientific resources) that curate high-quality, evidence-based medical information actually benefit from devoting funds and organizational resources to increase website user traffic and visibility via technical optimization (eg, site content and search engine compatibility) or by other means (eg, endorsement by influential people and use of slogans, catchphrases, logographic designs, and multimedia to enhance site awareness and recognition and to demonstrate expertise, authority, and trustworthiness).

Several tools were used to provide a robust quality assessment of the information encountered on various websites (ie, DISCERN, JAMA, and HON code of conduct). Although this approach is largely consistent with previous studies of web-based medical information, among these assessments, there are both redundant components (eg, authorship, attribution, and disclosure) as well as quality criteria that are unique to each of the tools used (eg, financial disclosure and advertising). Future studies may consider the use of more recent assessments comprising these and other essential criteria for evaluating the quality of information encountered on websites and web-based resources.

Additionally, the data extracted reflects publicly accessible information from the first 2 pages of returned results for 2 top trending search terms. Though not exhaustive (ie, all possible websites providing information on SCS), the search methodology used was intended to simulate scenario-specific consumer behavior (ie, seeking SCS-related information). Future studies may consider an alternate methodological approach involving divergent user-behavior scenarios.

### Conclusions

Publicly available web-based information on SCS mainly targets consumers and their caregivers. We found that almost all sites focused on surgically implanted SCS with less than half providing authorship details and publication dates. Most websites mentioned risks or side effects of SCS, yet few websites described possible contraindications. Sites curated by for-profit entities featured clinical or physician referral for SCS more often than other site types indicating implicit bias. This suggests that the public is likely to be exposed to incomplete, potentially misleading, or out-of-date information from unidentified sources regarding the use of SCS. For-profit sites were also encountered more frequently than nonprofit and scientific resource sites, suggesting the content and technical format of sites featuring higher-quality medical information are not optimized to enhance user traffic and search engine visibility. To avoid the spread of misinformation that could potentially harm certain consumer or patient groups, sites featuring evidence-based information on the current use of SCS should declare authorship, ensure the evidence is frequently updated, and consider using search engine optimization techniques that enable preferential site positioning for commonly used search terms.

## Appendix

Multimedia Appendix 1 Supplementary figures and tables.

## Abbreviations

ESCS: epidural spinal cord stimulation
HON: Health on the Net Foundation
JAMA: Journal of the American Medical Association
SCS: spinal cord stimulation
