# Supplementary material for: Web-Based Information on Spinal Cord Stimulation: Qualitative Assessment of Publicly Accessible Online Resources
Source: JMIR Public Health Surveill. 2024 Feb 23;10:e50031. doi: 10.2196/50031 (PMC10924266; doi:10.2196/50031)
Supplement: Multimedia Appendix 1 [file publichealth_v10i1e50031_app1.docx]

***Search strategy***

This study was prospectively registered in Open Science Framework (OSF) [28]. A systematic search was performed to identify websites curated by scientific resources (e.g., academic institutions and government organizations), non-profit/ foundations/ advocacy organizations, for-profit/private sector/industry, news/ media, individuals (e.g., blogs), and host sites for peer-reviewed journal abstracts and articles (e.g., Science Direct, Sage, Taylor & Francis online, etc.) which provide SCS-related information. All sites and advertisements without relevance to SCS were excluded.

A pilot search was conducted to identify the most popular terms searched (worldwide) within the last decade (December 28, 2012 to December 28, 2022) for the topic “spinal cord stimulator” using Google Trends. This approach is substantiated by exponential growth in the use of this index for evaluating population-specific user behavior and gaining insight on public health-related phenomena [33]. For this topic, the highest trending search queries (i.e., terms or phrases) identified were “spinal stimulator” and “spinal cord stimulator”. An advisory consortium of 3 individuals who had undergone epidural SCS implantation surgery was also consulted to validate the likelihood of using one or both search terms. The two terms were then entered in the four most popular search engines: Google (<https://www.google.com>), Baidu (<http://www.baidu.com>), Bing (<http://www.bing.com>), and Yahoo (<https://www.yahoo.com>) were used based on worldwide site ranking (<https://www.similarweb.com/top-websites/>). Baidu was used in conjunction with the Google Translate extension in the Google Chrome browser. At the time the search was conducted, Google, Baidu, Yahoo and Bing were ranked 1^st^, 6^th^, 9^th^ and 28^th^, respectively. Regional search interest for the topic “spinal cord stimulator” indicated the United States (100) [followed by Ireland (53), Laos (45), Australia (45), United Kingdom (30)] had the highest proportion of all queries for this topic within the specified timeframe (Figure 1). Based on these results, all searches were performed using a U.S. Internet Protocol (IP) designation to standardize the country-specific origin of returned results for each search engine [17]. Each search was performed in a new Incognito Window on the Google Chrome browser platform, to ensure previous search history and tracked cookies (i.e., browser cache) would not influence the search results. Duplicate websites encountered across search engines were noted (Table 1). Webpages were imported as PDF to Nvivo software (version 12, QRS International Pty Ltd, Doncaster, Australia) and archived for further analysis using the NCapture extension in Google Chrome.

***Quality and readability assessments***

The quality of the information provided on each website was assessed by two independent authors using the DISCERN instrument [29], the Journal of the American Medical Association (JAMA) benchmark criteria [30] and Health on the Net Foundation (HON) code of conduct certification [31]. The DISCERN instrument is a 16 item questionnaire evaluating information reliability (i.e., section 1, items 1-8), and the provision of information relating to treatment choices (e.g., active, self-care, no treatment, etc.) (i.e., section 2, items 9-15) with an additional overall quality rating (i.e., section 3, item 16). Items are rated on a Likert scale (i.e., 1=no, 2-4=partially, 5=yes). Ratings of high (≥4), moderate (3), or low (≤2) overall quality are given based on the majority of ratings for items 1-15. The instrument has demonstrated acceptable interrater agreement (i.e., Fleiss' kappa ≥0.40) [34]. JAMA benchmarks assessment is a 5-item tool used to evaluate the quality, transparency and reliability of online medical information based on 4 components (benchmark 1=authorship, benchmark 2=attribution, benchmark 3=currency, benchmark 4=disclosure). Each benchmark is scored according to a set of criteria (i.e., benchmark addressed=1, partially addressed=0.5, not addressed=0) which are summed to produce a total score ranging from 0-4 [30]. HON is an independent organization that evaluates the provision of reliable, transparent and ethical health information on publically accessible websites. The HON code of conduct is predicated on 8 principles (i.e., 1=Authority, 2=Complementarity, 3=Confidentiality, 4=Attribution, 5=Justifiability, 6=Transparency, 7=Financial disclosure, 8=Advertising). Presence or absence of the HON code of conduct certificate for each website was scored dichotomously (yes=1, no=0) [31].

Readability for each site was determined using the Flesch-Kincaid indices [32]. The Flesch-Kincaid indices provide estimates of reading ease [i.e., 206.835 - (1.015 x average sentence length) - (84.6 x average number of syllables per word] and the level of education required by the reader to understand the text on the first attempt (i.e., (0.39 x average sentence length) + (11.8 x average number of syllables per word) - 15.59). For reading ease, scores ranging from 0-30, 31-50, 51-60, 61-70, 71-80, 81-90, 90-100 indicated reading ease was very difficult, difficult, fairly difficult, standard, fairly easy, easy, or very easy, respectively. Grade level corresponds to standard US school grades (e.g., grade 9 indicates the first year of high school) [35]. For sites featuring only video content, audio to text captioning was used to extract text content for further analysis.

**Figure S1.** Word cloud & word frequency summary.

| **All websites** | **Word** | **Word Length** | **Frequency (Count)** | **Weighted Percentage (%)** |
| --- | --- | --- | --- | --- |
| **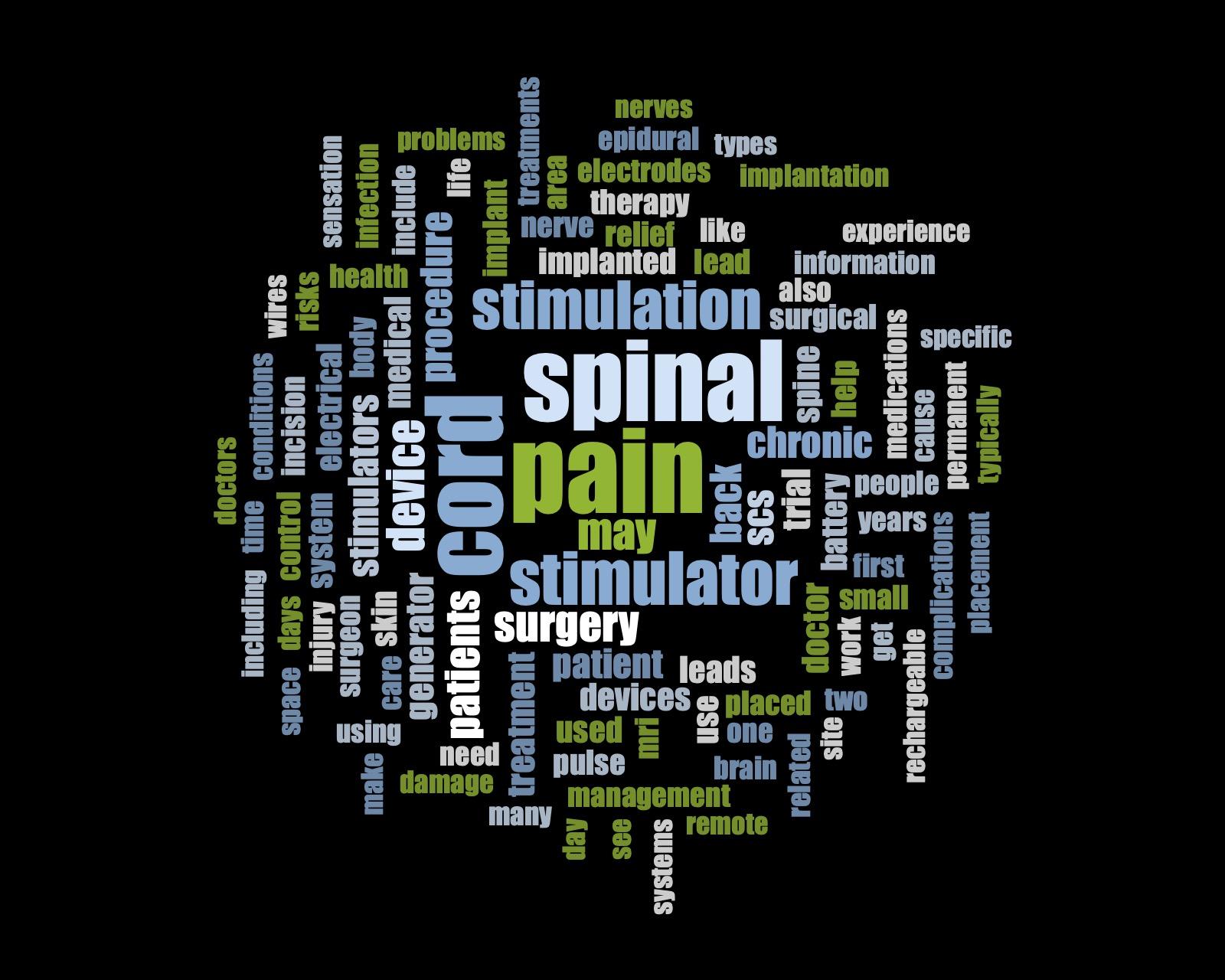** | pain | 4 | 763 | 2.95% |
|  | spinal | 6 | 720 | 2.78% |
|  | cord | 4 | 677 | 2.61% |
|  | stimulator | 10 | 394 | 1.52% |
|  | stimulation | 11 | 343 | 1.32% |
|  | device | 6 | 224 | 0.87% |
|  | may | 3 | 202 | 0.78% |
|  | surgery | 7 | 197 | 0.76% |
|  | patients | 8 | 184 | 0.71% |
|  | back | 4 | 149 | 0.58% |
|  | | | | |

A word cloud summary is shown above (left) with larger words indicating greater frequency relative to others. Words with the greatest overall frequency are also tabulated according to word length, total count and relative weight (right).

**Table S1.** Search term word frequency & readability of website information.

| **Type/ Title** | **Search Term Word Frequency** | | | | | | **Flesch-Kincaid** | |
| --- | --- | --- | --- | --- | --- | --- | --- | --- |
|  | **“Spinal”** | | **“Cord”** | | **“Stimulator”** | |  |  |
|  | **Count** | **%** | **Count** | **%** | **Count** | **%** | **Reading Ease** | **Grade** |
| **Scientific Resources (N=6)** |  |  |  |  |  |  |  |  |
| Spinal Cord Stimulator [36] | 46 | 4.58 | 45 | 4.48 | 34 | 3.39 | 49.2 | 8.5 |
| Spinal cord stimulation [37] | 38 | 1.69 | 29 | 1.29 | 14 | 0.62 | 57.7 | 7.7 |
| Back Pain and Spinal Cord Stimulation [38] | 11 | 6.32 | 11 | 6.32 | 0 | 0 | 59 | 6.4 |
| Treating Pain with Spinal Cord Stimulators [39] | 21 | 6.02 | 21 | 6.02 | 6 | 1.72 | 37.6 | 9.4 |
| Spinal Cord Stimulator Removal: Q&A with a Neurosurgeon [40] | 33 | 4.68 | 31 | 4.40 | 25 | 3.55 | 49.8 | 8.3 |
| Spinal Cord Stimulator Trial and Implantation [41] | 5 | 1.12 | 5 | 1.12 | 4 | 0.89 | 29.5 | 11.5 |
| **Non-Profit (N=12)** |  |  |  |  |  |  |  |  |
| Disadvantages and Risks of Spinal Cord Stimulation [42] | 14 | 3.57 | 14 | 3.57 | 0 | 0 | 48.3 | 8 |
| Spinal Cord Stimulation [43] | 8 | 0.88 | 8 | 0.88 | 3 | 0.33 | 54.8 | 8 |
| Spinal Cord Stimulation (Video Animation) [44] | 4 | 1.65 | 4 | 1.65 | 0 | 0 | 50 | 7 |
| Are Spinal Cord Stimulators Safe? What You Need to Know! [45] | 33 | 7.24 | 32 | 7.02 | 21 | 4.61 | 50.2 | 7.8 |
| Spinal cord stimulator [46] | 45 | 2.16 | 44 | 2.11 | 16 | 0.77 | 42.1 | 9 |
| Senza Spinal Cord Stimulation (SCS) System – P130022/S042 [47] | 5 | 1.9 | 5 | 1.9 | 1 | 0.38 | 51.6 | 7.7 |
| Spinal Cord Stimulation: Risks and Benefits [48] | 9 | 3.04 | 8 | 2.7 | 8 | 2.7 | 53.8 | 7.3 |
| Spinal Cord Stimulation for Chronic Back Pain Video [49] | 9 | 4.64 | 9 | 4.64 | 1 | 0.52 | 53.5 | 7 |
| Spinal Cord Stimulation for Chronic Back and Neck Pain [50] | 27 | 5.36 | 26 | 5.16 | 1 | 0.20 | 48.4 | 8.1 |
| Spinal cord stimulation: Placement and management [51] | 4 | 1.7 | 6 | 2.55 | 1 | 0.43 | 70.8 | 5.9 |
| Spinal cord stimulation [52] | 4 | 0.57 | 3 | 0.42 | 3 | 0.42 | 60.8 | 9.2 |
| Spinal Cord Stimulation's Role in Managing Chronic Disease PDF Symptoms [53] | 10 | 0.75 | 8 | 0.6 | 3 | 0.22 | 42 | 10.4 |
| **For-Profit (N=20)** |  |  |  |  |  |  |  |  |
| Spinal Cord Stimulator (SCS) Systems [54] | 24 | 4.56 | 24 | 4.56 | 24 | 4.56 | 36.5 | 13.4 |
| A NON-OPIOID ALTERNATIVE [55] | 4 | 3.6 | 4 | 3.6 | 1 | 0.9 | 33.5 | 11.3 |
| SPINAL CORD STIMULATION [56] | 21 | 2.41 | 18 | 2.06 | 3 | 0.34 | 59.7 | 7.3 |
| Spinal Cord Stimulation (a) [57] | 7 | 1.78 | 7 | 1.78 | 8 | 2.03 | 52.3 | 7.8 |
| Spinal Cord Stimulator Surgery – The risks and recovery time [58] | 16 | 3.23 | 15 | 3.02 | 17 | 3.43 | 54.6 | 8 |
| Spinal Cord Stimulation (SCS) [59] | 12 | 2.34 | 10 | 1.95 | 7 | 1.37 | 44.9 | 8.4 |
| Spinal Cord Stimulation Implant [60] | 10 | 7.30 | 9 | 6.57 | 6 | 4.38 | 45.9 | 7.9 |
| THE NEED FOR SPINAL CORD STIMULATION [61] | 11 | 2.24 | 11 | 2.24 | 8 | 1.63 | 47.4 | 11 |
| Spinal Cord Stimulator [62] | 8 | 3.59 | 8 | 3.59 | 5 | 2.24 | 45.5 | 8.5 |
| Spinal Cord Stimulators: Procedure and Recovery [63] | 9 | 3.2 | 9 | 3.2 | 3 | 1.07 | 56.9 | 6.9 |
| Spinal Cord Stimulator [64] | 20 | 5.10 | 20 | 5.10 | 8 | 2.04 | 36.3 | 10.2 |
| SPINAL CORD STIMULATION SYSTEMS HEALTHCARE PROFESSIONALS [65] | 1 | 0.43 | 1 | 0.43 | 0 | 0 | 19.3 | 12.2 |
| What You Need to Know About Spinal Cord Stimulator Surgery [66] | 33 | 2.86 | 27 | 2.34 | 16 | 1.39 | 52.6 | 7.5 |
| Spinal Cord Stimulator Review: Disadvantages And Risks Of The Surgery Implant [67] | 79 | 6.38 | 69 | 5.57 | 48 | 3.88 | 37.1 | 9 |
| Pain Management with Spinal Cord Stimulation Therapy [68] | 20 | 3.45 | 20 | 3.45 | 3 | 0.52 | 49.9 | 9.6 |
| Spinal Cord Stimulation - Surgical Procedure [69] | 5 | 0.95 | 5 | 0.95 | 2 | 0.38 | 62 | 8.8 |
| Spinal Cord Stimulator Trial / Implants [70] | 13 | 1.65 | 11 | 1.39 | 6 | 0.76 | 47.9 | 11.5 |
| The Next Era of Personalization: WaveWriter Alpha™ SCS System [71] | 9 | 0.53 | 9 | 0.53 | 24 | 1.41 | 38.1 | 11.8 |
| Types of Spinal Cord Stimulators: Functions, Differences, & Prices [72] | 11 | 2.24 | 11 | 2.24 | 8 | 1.63 | 36.5 | 8.9 |
| Living With A Spinal Cord Stimulator: Answers To FAQs [73] | 60 | 5.99 | 60 | 5.99 | 42 | 4.19 | 57.6 | 7.7 |
| **News/ Media (N=2)** |  |  |  |  |  |  |  |  |
| Spine Stimulator for Pain [74] | 1 | 0.52 | 1 | 0.52 | 1 | 0.52 | 32 | 21.7 |
| Spinal-cord stimulators help some patients, injure others [75] | 23 | 1.18 | 20 | 10.2 | 17 | 0.87 | 55.6 | 8.6 |
| **Personal/ blog (N=2)** |  |  |  |  |  |  |  |  |
| Anyone tried Spinal Cord Stimulation for Chronic Pain? [76] | 2 | 2.5 | 2 | 2.5 | 1 | 1.25 | 74.1 | 4.2 |
| What is a Spinal Cord Stimulator? [77] | 20 | 6.33 | 16 | 5.06 | 18 | 5.70 | 45.9 | 9.2 |

Flesch-Kincaid Reading Ease scores: 0-30=very difficult, 31-50=difficult, 51-60=fairly difficult, 61-70=standard, 71-80=fairly easy, 81-90=easy, 90-100=very easy.

Flesch-Kincaid Grade levels correspond to standard US school grades (e.g., grade 9 indicates the first year of high school).

**Figure S2.** Website quality summary.

| 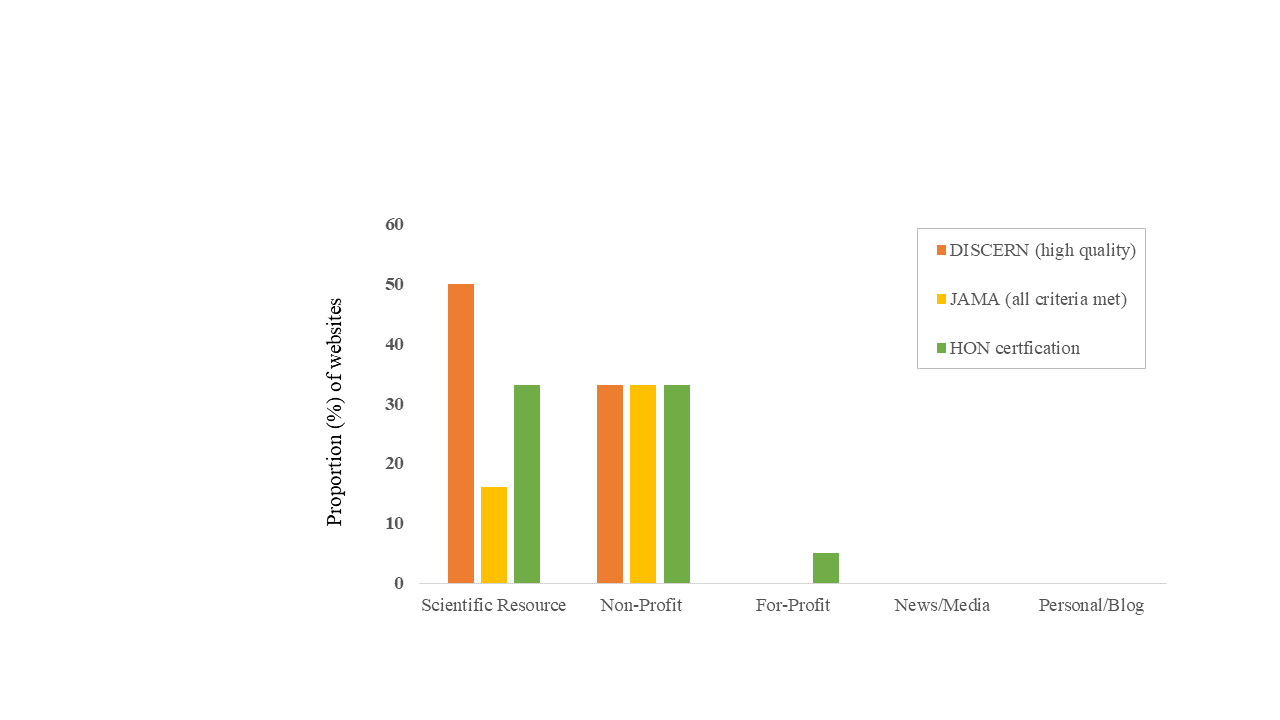 |
| --- |

A bar chart summarizing quality ratings according to website type is depicted above. The proportion of websites with high overall DICERN scores (≥4) and JAMA benchmark totals (4) was greater for scientific resource and non-profit site types. The proportion of websites with HON code certification was also greater for scientific resource and non-profit site types.

**Table S2.** Quality of website information.

|  | **DISCERN** | | | | | | | | | | | | | | | | **JAMA Benchmarks** | | | | | | **HON**  **code** |
| --- | --- | --- | --- | --- | --- | --- | --- | --- | --- | --- | --- | --- | --- | --- | --- | --- | --- | --- | --- | --- | --- | --- | --- |
|  | **Section**  **1** | | | | | | | | **Section**  **2** | | | | | | | **Section 3** |  |  |  |  |  |  |  |
| **Type/ Title** | **1** | **2** | **3** | **4** | **5** | **6** | **7** | **8** | **9** | **10** | **11** | **12** | **13** | **14** | **15** | **16** | **1** | **2** | **3** | **4** | **Total** |  | |
| **Scientific Resources (N=6)** |  |  |  |  |  |  |  |  |  |  |  |  |  |  |  |  |  |  |  |  |  | |  |
| Spinal Cord Stimulator [36] | 5 | 5 | 5 | 1 | 1 | 5 | 3 | 4 | 5 | 5 | 5 | 5 | 5 | 3 | 3 | 4 | 1 | 0 | 1 | 0 | 2 | | 0 |
| Spinal cord stimulation [37] | 5 | 5 | 5 | 5 | 5 | 5 | 5 | 5 | 5 | 5 | 5 | 1 | 5 | 1 | 5 | 4 | 1 | 1 | 1 | 1 | 4 | | 1 |
| Back Pain and Spinal Cord Stimulation [38] | 5 | 5 | 5 | 1 | 3 | 5 | 3 | 3 | 4 | 5 | 1 | 1 | 3 | 1 | 3 | 3 | 1 | 0 | 1 | 0 | 2 | | 1 |
| Treating Pain with Spinal Cord Stimulators [39] | 5 | 5 | 5 | 1 | 1 | 4 | 1 | 3 | 5 | 5 | 5 | 1 | 5 | 5 | 3 | 3 | 0.5 | 0 | 0 | 0 | 0.5 | | 0 |
| Spinal Cord Stimulator Removal: Q&A with a Neurosurgeon [40] | 5 | 5 | 5 | 1 | 5 | 5 | 1 | 5 | 5 | 5 | 5 | 1 | 5 | 5 | 5 | 4 | 1 | 0 | 1 | 0 | 2 | | 0 |
| Spinal Cord Stimulator Trial and Implantation [41] | 5 | 5 | 3 | 1 | 1 | 1 | 1 | 2 | 5 | 5 | 1 | 1 | 5 | 1 | 2 | 2 | 0 | 0 | 1 | 0 | 1 | | 0 |
| **Non-Profit (N=12)** |  |  |  |  |  |  |  |  |  |  |  |  |  |  |  |  |  |  |  |  |  | |  |
| Disadvantages and Risks of Spinal Cord Stimulation [42] | 5 | 5 | 5 | 1 | 5 | 5 | 5 | 5 | 2 | 3 | 5 | 1 | 5 | 5 | 2 | 4 | 0.5 | 0 | 0.5 | 1 | 2 | | 1 |
| Spinal Cord Stimulation [43] | 5 | 5 | 5 | 1 | 1 | 5 | 2 | 3 | 3 | 5 | 1 | 5 | 5 | 5 | 4 | 4 | 1 | 0 | 1 | 1 | 3 | | 0 |
| Spinal Cord Stimulation (Video Animation) [44] | 1 | 1 | 1 | 1 | 1 | 1 | 1 | 1 | 1 | 1 | 1 | 1 | 1 | 1 | 1 | 2 | 0 | 0 | 1 | 0 | 1 | | 0 |
| Are Spinal Cord Stimulators Safe? What You Need to Know! [45] | 5 | 5 | 5 | 1 | 5 | 3 | 4 | 3 | 5 | 5 | 5 | 5 | 5 | 5 | 5 | 2 | 1 | 0 | 0.5 | 0.5 | 2 | | 0 |
| Spinal cord stimulator [46] | 5 | 5 | 5 | 5 | 5 | 5 | 5 | 5 | 5 | 5 | 5 | 5 | 5 | 5 | 5 | 4 | 1 | 1 | 1 | 1 | 4 | | 0 |
| Senza Spinal Cord Stimulation (SCS) System – P130022/S042 [47] | 5 | 5 | 5 | 1 | 5 | 1 | 5 | 3 | 1 | 3 | 3 | 1 | 1 | 1 | 1 | 4 | 1 | 0 | 1 | 0.5 | 2.5 | | 0 |
| Spinal Cord Stimulation: Risks and Benefits [48] | 5 | 5 | 5 | 1 | 5 | 1 | 1 | 1 | 2 | 5 | 5 | 1 | 5 | 1 | 1 | 3 | 1 | 0 | 1 | 0 | 2 | | 0 |
| Spinal Cord Stimulation for Chronic Back Pain Video [49] | 5 | 5 | 5 | 1 | 1 | 5 | 1 | 2 | 5 | 1 | 1 | 1 | 1 | 1 | 1 | 3 | 1 | 0 | 1 | 0 | 2 | | 1 |
| Spinal Cord Stimulation for Chronic Back and Neck Pain [50] | 5 | 5 | 5 | 1 | 1 | 5 | 3 | 1 | 5 | 5 | 1 | 1 | 5 | 2 | 3 | 3 | 1 | 1 | 1 | 0 | 3 | | 1 |
| Spinal cord stimulation: Placement and management [51] | 5 | 5 | 5 | 5 | 5 | 5 | 5 | 5 | 1 | 1 | 1 | 1 | 1 | 1 | 3 | 2 | 1 | 1 | 1 | 1 | 4 | | 0 |
| Spinal cord stimulation [52] | 5 | 5 | 5 | 5 | 5 | 5 | 3 | 1 | 5 | 5 | 5 | 1 | 5 | 1 | 4 | 3 | 1 | 1 | 1 | 0 | 3 | | 0 |
| Spinal Cord Stimulation's Role in Managing Chronic Disease PDF Symptoms [53] | 5 | 5 | 5 | 5 | 5 | 5 | 1 | 5 | 5 | 5 | 5 | 1 | 5 | 3 | 3 | 3 | 0.5 | 1 | 1 | 0.5 | 3 | | 1 |
| **For-Profit (N=20)** |  |  |  |  |  |  |  |  |  |  |  |  |  |  |  |  |  |  |  |  |  | |  |
| Spinal Cord Stimulator (SCS) Systems [54] | 5 | 1 | 5 | 1 | 1 | 1 | 5 | 2 | 1 | 1 | 1 | 1 | 1 | 4 | 1 | 2 | 0 | 0 | 0 | 0 | 0 | | 0 |
| A NON-OPIOID ALTERNATIVE [55] | 5 | 5 | 5 | 5 | 1 | 3 | 4 | 5 | 5 | 5 | 5 | 1 | 5 | 5 | 3 | 3 | 0 | 1 | 1 | 0.5 | 2.5 | | 0 |
| SPINAL CORD STIMULATION [56] | 5 | 5 | 5 | 1 | 1 | 4 | 2 | 4 | 5 | 5 | 5 | 5 | 5 | 5 | 5 | 3 | 0 | 0 | 1 | 0 | 1 | | 0 |
| Spinal Cord Stimulation (a) [57] | 5 | 5 | 5 | 1 | 1 | 2 | 1 | 3 | 3 | 5 | 3 | 5 | 5 | 1 | 2 | 3 | 0 | 0 | 1 | 0.5 | 1.5 | | 0 |
| Spinal Cord Stimulator Surgery – The risks and recovery time [58] | 5 | 5 | 5 | 1 | 5 | 1 | 1 | 3 | 4 | 5 | 5 | 2 | 5 | 1 | 5 | 3 | 0 | 0 | 1 | 0 | 1 | | 0 |
| Spinal Cord Stimulation (SCS) [59] | 5 | 5 | 5 | 1 | 5 | 3 | 5 | 4 | 5 | 5 | 1 | 1 | 4 | 1 | 3 | 2 | 0 | 0 | 1 | 1 | 2 | | 0 |
| Spinal Cord Stimulation Implant [60] | 5 | 5 | 5 | 1 | 1 | 1 | 1 | 2 | 4 | 5 | 1 | 1 | 1 | 1 | 4 | 2 | 0 | 0 | 0 | 0 | 0 | | 0 |
| THE NEED FOR SPINAL CORD STIMULATION [61] | 5 | 5 | 4 | 1 | 1 | 1 | 1 | 3 | 5 | 5 | 1 | 2 | 5 | 1 | 3 | 2 | 0 | 0 | 1 | 0 | 1 | | 0 |
| Spinal Cord Stimulator [62] | 5 | 5 | 5 | 1 | 1 | 1 | 1 | 3 | 5 | 5 | 1 | 1 | 5 | 1 | 3 | 3 | 0 | 0 | 1 | 0.5 | 1.5 | | 0 |
| Spinal Cord Stimulators: Procedure and Recovery [63] | 5 | 5 | 5 | 1 | 1 | 2 | 1 | 3 | 5 | 5 | 5 | 1 | 5 | 1 | 5 | 3 | 0.5 | 0 | 1 | 0 | 1.5 | | 0 |
| Spinal Cord Stimulator [64] | 5 | 5 | 5 | 1 | 1 | 1 | 3 | 3 | 5 | 1 | 5 | 1 | 1 | 5 | 5 | 2 | 0 | 0 | 1 | 0 | 1 | | 0 |
| SPINAL CORD STIMULATION SYSTEMS HEALTHCARE PROFESSIONALS [65] | 3 | 3 | 3 | 1 | 1 | 1 | 1 | 1 | 1 | 1 | 1 | 1 | 3 | 5 | 1 | 2 | 0 | 0 | 1 | 0.5 | 1.5 | | 0 |
| What You Need to Know About Spinal Cord Stimulator Surgery [66] | 5 | 5 | 5 | 1 | 1 | 1 | 1 | 3 | 5 | 5 | 5 | 1 | 5 | 1 | 3 | 3 | 0 | 0 | 1 | 0 | 1 | | 0 |
| Spinal Cord Stimulator Review: Disadvantages And Risks Of The Surgery Implant [67] | 5 | 5 | 5 | 1 | 1 | 5 | 2 | 5 | 5 | 5 | 5 | 5 | 5 | 5 | 3 | 3 | 1 | 0 | 1 | 0 | 2 | | 0 |
| Pain Management with Spinal Cord Stimulation Therapy [68] | 5 | 5 | 5 | 1 | 5 | 3 | 3 | 5 | 5 | 5 | 5 | 1 | 5 | 1 | 3 | 3 | 1 | 0 | 1 | 0.5 | 2.5 | | 0 |
| Spinal Cord Stimulation - Surgical Procedure [69] | 5 | 5 | 5 | 1 | 1 | 3 | 3 | 2 | 5 | 1 | 1 | 1 | 2 | 1 | 3 | 3 | 0 | 0 | 1 | 0 | 1 | | 0 |
| Spinal Cord Stimulator Trial / Implants [70] | 5 | 5 | 5 | 1 | 1 | 3 | 1 | 2 | 5 | 5 | 3 | 1 | 5 | 1 | 3 | 3 | 0 | 0 | 1 | 0 | 1 | | 1 |
| The Next Era of Personalization: WaveWriter Alpha™ SCS System [71] | 2 | 3 | 5 | 1 | 1 | 1 | 1 | 1 | 1 | 1 | 1 | 1 | 1 | 1 | 1 | 2 | 0 | 0 | 0.5 | 0 | 0.5 | | 0 |
| Types of Spinal Cord Stimulators: Functions, Differences, & Prices [72] | 5 | 5 | 5 | 1 | 1 | 1 | 1 | 1 | 5 | 5 | 1 | 1 | 5 | 1 | 3 | 2 | 0.5 | 0 | 1 | 0 | 1.5 | | 0 |
| Living With A Spinal Cord Stimulator: Answers To FAQs [73] | 5 | 5 | 5 | 1 | 1 | 2 | 1 | 1 | 5 | 5 | 1 | 1 | 5 | 5 | 3 | 2 | 1 | 0 | 1 | 0 | 2 | | 0 |
| **News/ Media (N=2)** |  |  |  |  |  |  |  |  |  |  |  |  |  |  |  |  |  |  |  |  |  | |  |
| Spine Stimulator for Pain [74] | 5 | 5 | 5 | 1 | 5 | 3 | 1 | 4 | 5 | 5 | 2 | 1 | 5 | 1 | 3 | 2 | 1 | 0 | 0.5 | 0 | 1.5 | | 0 |
| Spinal-cord stimulators help some patients, injure others [75] | 5 | 5 | 5 | 5 | 5 | 2 | 5 | 4 | 1 | 5 | 5 | 1 | 5 | 3 | 3 | 2 | 1 | 0 | 1 | 0.5 | 2.5 | | 0 |
| **Personal/ blog (N=2)** |  |  |  |  |  |  |  |  |  |  |  |  |  |  |  |  |  |  |  |  |  | |  |
| Anyone tried Spinal Cord Stimulation for Chronic Pain? [76] | 5 | 5 | 5 | 1 | 1 | 2 | 4 | 1 | 1 | 5 | 1 | 5 | 5 | 5 | 5 | 2 | 1 | 0 | 1 | 0 | 2 | | 0 |
| What is a Spinal Cord Stimulator? [77] | 5 | 5 | 5 | 1 | 1 | 4 | 1 | 3 | 5 | 5 | 1 | 1 | 5 | 1 | 1 | 3 | 1 | 0 | 1 | 0 | 2 | | 0 |

DISCERN (Scoring Items 1-15: 1=no, 2-4=partially, 5=yes) (Scoring Item 16: high (≥4), moderate (3), or low (≤2) overall quality rating)

Item 1: Are the aims clear?

Item 2: Does it achieve its aims?

Item 3: Is it relevant?

Item 4: Is it clear what sources of information were used to compile the publication (other than the author or producer)?

Item 5: Is it clear when the information used or reported in the publication was produced?

Item 6: Is it balanced and unbiased?

Item 7: Does it provide details of additional sources of support and information?

Item 8: Does it refer to areas of uncertainty?

Item 9: Does it describe how each treatment works?

Item 10: Does it describe the benefits of each treatment?

Item 11: Does it describe the risks of each treatment?

Item 12: Does it describe what would happen if no treatment is used?

Item 13: Does it describe how the treatment choices affect overall quality of life?

Item 14: Is it clear that there may be more than one possible treatment choice?

Item 15: Does it provide support for shared decision-making?

Item 16: Based on the answers to all of the above questions, rate the overall quality of the publication as a source of information about treatment choices.

JAMA Benchmarks (standards for Internet sources of medical information) (Scoring: benchmark addressed=1, partially addressed=0.5, not addressed=0)

Benchmark 1 (Authorship): proper citations used in the website

Benchmark 2 (Attribution): references and sources of information are identified

Benchmark 3 (Currency): the website is updated with the latest information

Benchmark 4 (Disclosure): website ownership, advertising, and conflicts of interest are disclosed.

HONcode Principles (Scoring: 1=Yes 0=No):

Principle 1 (Authority): Provide author qualifications

Principle 2 (Complementarity): Provide supporting information

Principle 3 (Confidentiality): Respect the privacy of site users

Principle 4 (Attribution): Cite the sources and dates of medical information

Principle 5 (Justifiability): Justification of claims/ balanced and objective claims

Principle 6 (Transparency): Accessibility, provide valid contact details

Principle 7 (Financial disclosure): Provide details of funding

Principle 8 (Advertising): Clearly distinguish advertising from editorial content

**Table S3.** Summary of website characteristics and information categories.

| Website Category | Scientific Resource (n=6) | Nonprofit (n=12) | For-Profit (n=20) | News/ Media (n=2) | Personal/ blog (n=2) |
| --- | --- | --- | --- | --- | --- |
| Website Characteristics |  |  |  |  |  |
|  |  |  |  |  |  |
| Author/ credentials | 4 (66) | 9 (75) | 3 (15) | 2 (100) | 2 (100) |
| Publication date | 2 (33) | 8 (66) | 5 (25) | 2 (100) | 2 (100) |
| Geographical location/ designation | 5 (83) | 6 (50) | 19 (95) | 2 (100) | 1 (50) |
| Accessibility | 4 (66) | 6 (50) | 7 (35) | 2 (100) | 1 (50) |
| Pop-ups/ notification/ advertisements | 1 (16) | 1 (5) | 6 (30) | 0 (0) | 1 (50) |
| Peer-reviewed references | 1 (16) | 6 (50) | 1 (5) | 0 (0) | 0 (0) |
| Number of peer-reviewed references | 1.17±2.86 | 11.08±27.05 | 0.10±0.45 | 0.0±0.0 | 0.0±0.0 |
|  |  |  |  |  |  |
| Website Information Type |  |  |  |  |  |
|  |  |  |  |  |  |
| 1. SCS Definition/ Summary |  |  |  |  |  |
| Summary statement provided | 5 (83) | 12 (100) | 17 (85) | 2 (100) | 1 (50) |
| SCS definition provided | 5 (83) | 11 (91) | 16 (80) | 2 (100) | 1 (50) |
| How SCS is implanted | 5 (83) | 12 (100) | 16 (80) | 2 (100) | 1 (50) |
| Who performs the procedure | 3 (50) | 5 (41) | 11 (55) | 2 (100) | 0 (0) |
| 2. SCS Type |  |  |  |  |  |
| ESCS | 5 (83) | 10 (83) | 18 (90) | 2 (100) | 2 (100) |
| TSCS | 0 (0) | 0 (0) | 0 (0) | 0 (0) | 0 (0) |
| Both | 1 (16) | 0 (0) | 1 (5) | 0 (0) | 0 (0) |
| SCS subcategory (implantable pulse generator, radiofrequency stimulator) | 4 (66) | 4 (33) | 6 (30) | 0 (0) | 0 (0) |
| 3. Indications |  |  |  |  |  |
| Indications described | 5 (83) | 12 (100) | 18 (90) | 2 (100) | 1 (50) |
| Target population | 4 (66) | 10 (83) | 18 (90) | 2 (100) | 1 (50) |
| Specific symptoms | 4 (66) | 10 (83) | 18 (90) | 2 (100) | 1 (50) |
| Screening or evaluation for eligibility | 2 (33) | 7 (58) | 13 (65) | 1 (50.0) | 1 (50) |
| 3.1 FDA Approved Indications | 5 (83) | 12 (100) | 18 (90) | 2 (100) | 1 (50) |
| Chronic intractable trunk/limb pain | 5 (83) | 12 (100) | 18 (90) | 2 (100) | 1 (50) |
| Failed back surgery syndrome | 3 (50) | 3 (25) | 9 (45) | 0 (0) | 0 (0) |
| Complex regional pain syndrome | 3 (50) | 4 (33) | 11 (55) | 0 (0) | 0 (0) |
| Intractable low back pain and leg pain | 0 (0) | 7 (58) | 9 (45) | 1 (50) | 0 (0) |
| Radicular pain syndrome | 1 (16) | 1 (8) | 1 (5) | 0 (0) | 0 (0) |
| Radiculopathies (pain secondary to failed back syndrome or herniated disc) | 1 (16) | 1 (8) | 4 (20) | 0 (0) | 0 (0) |
| Epidural fibrosis | 0 (0) | 0 (0) | 1 (5) | 0 (0) | 0 (0) |
| Degenerative disc disease (pain refractory to (non)surgical interventions) | 0 (0) | 0 (0) | 2 (10) | 0 (0) | 0 (0) |
| Arachnoiditis | 3 (50) | 1 (8) | 7 (35) | 0 (0) | 0 (0) |
| Multiple back surgeries | 3 (50) | 0 (0) | 1 (5) | 0 (0) | 0 (0) |
| 3.2 Other Indications | 4 (66) | 2 (16) | 8 (40) | 0 (0) | 0 (0) |
| Intractable angina pectoris | 3 (50) | 2 (16) | 5 (25) | 0 (0) | 0 (0) |
| Peripheral vascular disease | 3 (50) | 0 (0) | 8 (40) | 0 (0) | 0 (0) |
| Spinal cord injury | 3 (50) | 0 (0) | 5 (25) | 0 (0) | 0 (0) |
| Cancer-related pain | 5 (83) | 0 (0) | 1 (5) | 0 (0) | 1 (50) |
| Perineal pain | 2 (33) | 1 (8) | 2 (10) | 0 (0) | 0 (0) |
| Migraines | 0 (0) | 0 (0) | 1 (5) | 0 (0) | 1 (50) |
| Shingles | 0 (0) | 0 (0) | 1 (5) | 0 (0) | 0 (0) |
| Diabetic neuropathy | 4 (66) | 2 (16) | 5 (25) | 0 (0) | 0 (0) |
| 3.3 Other Symptoms/ Conditions | 5 (83) | 2 (16) | 9 (45) | 0 (0) | 1 (50) |
| Type of pain described | 5 (83) | 12 (100) | 18 (90) | 2 (100) | 1 (50) |
| Chronic pain | 5 (83) | 12 (100) | 18 (90) | 2 (100) | 1 (50) |
| Radicular pain | 1 (16) | 1 (8) | 1 (5) | 0 (0) | 0 (0) |
| Visceral pain | 2 (33) | 2 (16) | 2 (10) | 0 (0) | 0 (0) |
| Musculoskeletal pain | 0 (0) | 1 (8) | 0 (0) | 0 (0) | 0 (0) |
| Neuropathic pain | 4 (66) | 4 (33) | 7 (35) | 0 (0) | 0 (0) |
| Amputation-related pain | 3 (50) | 0 (0) | 5 (25) | 0 (0) | 0 (0) |
| Perineal pain | 2 (33) | 1 (8) | 2 (10) | 0 (0) | 0 (0) |
| Complex regional pain syndrome | 3 (50) | 4 (33) | 11 (55) | 0 (0) | 0 (0) |
| 4. Contraindications |  |  |  |  |  |
| Contraindications described | 1 (16) | 4 (33) | 8 (40) | 0 (0) | 1 (50) |
| FDA approved contraindications | 1 (16) | 3 (25) | 5 (25) | 0 (0) | 1 (50) |
| Poor surgical candidates | 0 (0) | 3 (25) | 4 (20) | 0 (0) | 1 (50) |
| Unable to operate SCS system | 0 (0) | 1 (8) | 2 (10) | 0 (0) | 0 (0) |
| No pain relief during the trial | 1 (16) | 1 (8) | 3 (15) | 0 (0) | 0 (0) |
| Pregnancy | 0 (0) | 0 (0) | 2 (10) | 0 (0) | 0 (0) |
| Implants (metal implants and/or baclofen pump) | 0 (0) | 0 (0) | 1 (5) | 0 (0) | 0 (0) |
| Pacemakers | 0 (0) | 2 (16) | 3 (15) | 0 (0) | 1 (50) |
| Pacemaker explicitly stated as contraindication | 0 (0) | 2 (16) | 0 (0) | 0 (0) | 1 (50) |
| Psychological disorders | 1 (16) | 1 (8) | 2 (10) | 0 (0) | 1 (50) |
| Other contraindications | 0 (0) | 1 (8) | 1 (5) | 0 (0) | 1 (50) |
| 5. Side Effects/ Risks |  |  |  |  |  |
| Side effects or risks described | 4 (66) | 6 (50) | 16 (80) | 1 (50) | 1 (50) |
| Infection | 3 (50) | 6 (50) | 10 (50) | 0 (0) | 0 (0) |
| Trauma, bleeding, or puncture to the spinal cord or other tissue | 2 (33) | 6 (50) | 8 (40) | 0 (0) | 1 (50) |
| Device migration, damage, or malfunction | 3 (50) | 6 (50) | 9 (45) | 1 (50) | 0 (0) |
| Side effect frequency, likelihood, and severity | 1 (16) | 4 (33) | 4 (20) | 1 (50) | 0 (0) |
| Restrictions (MRI, showering, wheelchair use) | 2 (33) | 4 (33) | 10 (50) | 0 (0) | 1 (50) |
| Indication device is MRI-compatible or some models may be MRI-compatible | 2 (33) | 3 (25) | 5 (25) | 0 (0) | 0 (0) |
| Other risks | 2 (33) | 5 (41) | 9 (45) | 0 (0) | 1 (50) |
| 6. Device Considerations |  |  |  |  |  |
| Device considerations stated | 6 (100) | 6 (50) | 12 (60) | 0 (0) | 1 (50) |
| Battery life in general | 5 (83) | 5 (41) | 10 (50) | 0 (0) | 0 (0) |
| Estimated battery life | 1 (16) | 4 (33) | 6 (30) | 0 (0) | 0 (0) |
| Required maintenance | 0 (0) | 0 (0) | 5 (25) | 0 (0) | 0 (0) |
| Alternative treatment options | 2 (33) | 1 (8) | 3 (15) | 0 (0) | 0 (0) |
| Other considerations | 0 (0) | 1 (8) | 2 (10) | 0 (0) | 1 (50) |
| 7. Follow-up |  |  |  |  |  |
| Follow-up described | 2 (33) | 5 (41) | 14 (70) | 1 (50) | 0 (0) |
| Treatment or recovery pathway/protocol | 2 (33) | 2 (16) | 12 (60) | 1 (50) | 0 (0) |
| Assessments | 2 (33) | 2 (16) | 1 (5) | 0 (0) | 0 (0) |
| Pre-operative consumer education | 5 (83) | 12 (100) | 18 (90) | 2 (100) | 1 (50) |
| Post-operative consumer education | 2 (33) | 3 (25) | 10 (50) | 0 (0) | 0 (0) |
| Training required | 1 (16) | 0 (0) | 0 (0) | 0 (0) | 0 (0) |
| 8. Outcomes |  |  |  |  |  |
| Expected outcomes stated | 3 (50) | 8 (66) | 18 (90) | 2 (100) | 0 (0) |
| Treatment efficacy (restoring function, reducing symptoms) | 3 (50) | 8 (66) | 17 (85) | 2 (100) | 0 (0) |
| Satisfaction (consumer testimonials, quotes) | 1 (16) | 0 (0) | 2 (10) | 2 (100) | 0 (0) |
| 9. Intended Audience |  |  |  |  |  |
| Clinicians & researchers | 0 (0) | 0 (0) | 1 (5) | 0 (0) | 0 (0) |
| Consumers & caregivers | 3 (50) | 7 (58) | 15 (75) | 0 (0) | 2 (100) |
| Both | 3 (50) | 5 (41) | 4 (20) | 2 (100) | 0 (0) |
| 10. Referral |  |  |  |  |  |
| Physician/ clinic referral advertised | 3 (50) | 7 (58) | 17 (85) | 0 (0) | 0 (0) |

All data are reported as frequencies (n) and proportions (%), or mean±sd.

Abbreviations: ESCS=epidural spinal cord stimulation, FDA=Food and Drug Administration, MRI=magnetic resonance imaging, TSCS=transcutaneous spinal cord stimulation

**Table S4.** Website characteristics.

| **Title** | **Author/ Credentials** | **Publication/ Last Update** | **Country/ Designation** | **Accessibility Options** | **Notification/ Pop-up/ Advertisement** | **Number of References** |
| --- | --- | --- | --- | --- | --- | --- |
| **Scientific Resources (N=6)** |  |  |  |  |  |  |
| Spinal Cord Stimulator [36] | Eellan Sivanesan, M.D. | No Date | USA | Website, Video, FAQ | No | 0 |
| Spinal cord stimulation [37] | Tann Nichols, MD; George Mandybur, MD; Marc Orlando, MD | 07/2021 | USA | Website, PDF, Video, Skype Number | No | 7 |
| Back Pain and Spinal Cord Stimulation [38] | No Author | 02/05/2020 | No Location | Website | Yes | 0 |
| Treating Pain with Spinal Cord Stimulators [39] | No Author | No Date | USA | Website | No | 0 |
| Spinal Cord Stimulator Removal: Q&A with a Neurosurgeon [40] | William S Anderson, M.A., M.D., Ph.D. | No Date | USA | FAQ | No | 0 |
| Spinal Cord Stimulator Trial and Implantation [41] | UTH health | No Date | USA | Website, Referral | No | 0 |
| **Non-Profit (N=12)** |  |  |  |  |  |  |
| Disadvantages and Risks of Spinal Cord Stimulation [42] | Neel Mehta, MD | 09/23/2016 | No Location | Website, Video | No | 5 |
| Spinal Cord Stimulation [43] | Brian Anderson; Kevin M. Cockroft, MD, FAANS | No Date | USA | Website | No | 0 |
| Spinal Cord Stimulation (Video Animation) [44] | No Author | No Date | No Location | Video | No | 0 |
| Are Spinal Cord Stimulators Safe? What You Need to Know! [45] | Spinal Cord.com team | 12/03/2020 | USA | Website, Testimonials | Yes | 0 |
| Spinal cord stimulator [46] | Wikipedia | 03/22/2022 | No Location | Website, Images | No | 25 |
| Senza Spinal Cord Stimulation (SCS) System – P130022/S042 [47] | No Author | 02/15/2022 | USA | Website | No | 0 |
| Spinal Cord Stimulation: Risks and Benefits [48] | Lawrence M. Kamhi, MD, FIPP | 09/12/2017 | No Location | Website | No | 0 |
| Spinal Cord Stimulation for Chronic Back Pain Video [49] | Veritas Health | No Date | No Location | Video | No | 0 |
| Spinal Cord Stimulation for Chronic Back and Neck Pain [50] | Neel Mehta, MD | 09/23/2016 | No Location | Website, Video | No | 5 |
| Spinal cord stimulation: Placement and management [51] | Anne Marie McKenzie-Brown, MD; Scott G Pritzlaff, MD | 1/11/2022 | USA | Website | No | 94 |
| Spinal cord stimulation [52] | No Author | No Date | USA | Website | No | 3 |
| Spinal Cord Stimulation's Role in Managing Chronic Disease PDF Symptoms [53] | Dr. Simon Thomson | 12/13/2019 | USA | Website | No | 1 |
| **For-profit (N=20)** |  |  |  |  |  |  |
| Spinal Cord Stimulator (SCS) Systems [54] | No Author | No Date | USA | Website, Video, FAQ | No | 0 |
| A NON-OPIOID ALTERNATIVE [55] | No Author | No Date | No Location | Website, Video, Pop-up | Yes | 2 |
| SPINAL CORD STIMULATION [56] | No Author | No Date | Australia | Website, Video, PDF | No | 0 |
| Spinal Cord Stimulation (a) [57] | No Author | No Date | USA | Website | No | 0 |
| Spinal Cord Stimulator Surgery – The risks and recovery time [58] | No Author | 05/04/2017 | USA | Website | No | 0 |
| Spinal Cord Stimulation (SCS) [59] | No Author | No Date | USA | Website, Video | No | 0 |
| Spinal Cord Stimulation Implant [60] | No Author | No Date | USA | Website | Yes | 0 |
| THE NEED FOR SPINAL CORD STIMULATION [61] | No Author | No Date | USA | Website, Video, Patient FAQ | Yes | 0 |
| Spinal Cord Stimulator [62] | No Author | No Date | USA | Website, Video | No | 0 |
| Spinal Cord Stimulators: Procedure and Recovery [63] | Lawrence M. Kamhi, MD, FIPP | 10/06/2021 | No Location | Website | Yes | 0 |
| Spinal Cord Stimulator [64] | No Author | No Date | USA | Website, | No | 0 |
| SPINAL CORD STIMULATION SYSTEMS HEALTHCARE PROFESSIONALS [65] | No Author | 06/2021 | USA | Website | No | 0 |
| What You Need to Know About Spinal Cord Stimulator Surgery [66] | No Author | No Date | USA | Website | Yes | 0 |
| Spinal Cord Stimulator Review: Disadvantages And Risks Of The Surgery Implant [67] | Dr. Deuk | No Date | USA | Website, Videos | No | 0 |
| Pain Management with Spinal Cord Stimulation Therapy [68] | No Author | 01/28/2022 | USA | Website | No | 0 |
| Spinal Cord Stimulation - Surgical Procedure [69] | No Author | No Date | Canada | Website | No | 0 |
| Spinal Cord Stimulator Trial / Implants [70] | No Author | No Date | USA | Website | No | 0 |
| The Next Era of Personalization: WaveWriter Alpha™ SCS System [71] | No Author | No Date | USA | Website | Yes | 0 |
| Types of Spinal Cord Stimulators: Functions, Differences, & Prices [72] | No Author | No Date | USA | Website | No | 0 |
| Living With A Spinal Cord Stimulator: Answers To FAQs [73] | Arizonapain | 07/13/2020 | USA | Website | No | 0 |
| **News/ Media (N=2)** |  |  |  |  |  |  |
| Spine Stimulator for Pain [74] | Mayo Clinic | 09/15/2015 | USA | Youtube Video | No | 0 |
| Spinal-cord stimulators help some patients, injure others [75] | Mitch Weiss; Holbrook Mohr | 11/28/2018 | USA | Website, Video | No | 0 |
| **Personal/ blog (N=2)** |  |  |  |  |  |  |
| Anyone tried Spinal Cord Stimulation for Chronic Pain? [76] | steeldove | 10/25/2018 | USA | Website, Q&A | Yes | 0 |
| What is a Spinal Cord Stimulator? [77] | Erin Oxendine | 05/06/2022 | no location | Website | No | 0 |

Search engine: Google=1, Baidu=2, Yahoo=3, Bing=4

Abbreviations: N/A=Not Applicable

**Table S5.** Website information categories.

| **Title/ Reference** | **Information Category** | | | | | | | | | | |
| --- | --- | --- | --- | --- | --- | --- | --- | --- | --- | --- | --- |
|  | **1** | **2** | | **3** | **4** | **5** | **6** | **7** | **8** | **9^†^** | **10** |
|  |  | SCS Type | Subcategory |  |  |  |  |  |  |  |  |
| **Scientific Resources (N=6)** |  |  |  |  |  |  |  |  |  |  |  |
| Spinal Cord Stimulator [36] | Yes | ESCS | Yes | Yes | Yes | Yes | Yes | Yes | Yes | Both | No |
| Spinal cord stimulation [37] | Yes | ESCS | Yes | Yes | No | Yes | Yes | Yes | Yes | Both | No |
| Back Pain and Spinal Cord Stimulation [38] | Yes | ESCS | Yes | Yes | No | Yes | No | No | No | Consumers/ Caregivers | No |
| Treating Pain with Spinal Cord Stimulators [39] | Yes | ESCS/ TSCS | Yes | Yes | No | Yes | No | No | No | Both | Yes |
| Spinal Cord Stimulator Removal: Q&A with a Neurosurgeon [40] | No | ESCS | No | No | Yes | Yes | No | No | No | Consumers/ Caregivers | Yes |
| Spinal Cord Stimulator Trial and Implantation [41] | Yes | ESCS | No | Yes | No | No | Yes | No | Yes | Consumers/ Caregivers | Yes |
| **Non-profit (N=12)** |  |  |  |  |  |  |  |  |  |  |  |
| Disadvantages and Risks of Spinal Cord Stimulation [42] | Yes | Undefined | Yes | Yes | No | Yes | Yes | No | Yes | Consumers/ Caregivers | Yes |
| Spinal Cord Stimulation [43] | Yes | ESCS | No | Yes | Yes | No | Yes | Yes | Yes | Both | Yes |
| Spinal Cord Stimulation (Video Animation) [44] | Yes | ESCS | No | Yes | No | No | No | Yes | No | Both | No |
| Are Spinal Cord Stimulators Safe? What You Need to Know! [45] | Yes | ESCS | No | Yes | No | Yes | No | No | Yes | Consumers/ Caregivers | Yes |
| Spinal cord stimulator [46] | Yes | ESCS | Yes | Yes | Yes | Yes | Yes | Yes | Yes | Both | No |
| Senza Spinal Cord Stimulation (SCS) System – P130022/S042 [47] | Yes | ESCS | No | Yes | Yes | No | No | No | No | Both | No |
| Spinal Cord Stimulation: Risks and Benefits [48] | Yes | ESCS | No | Yes | No | Yes | Yes | Yes | Yes | Consumers/ Caregivers | Yes |
| Spinal Cord Stimulation for Chronic Back Pain Video [49] | Yes | ESCS | No | Yes | No | No | No | No | No | Consumers/ Caregivers | No |
| Spinal Cord Stimulation for Chronic Back and Neck Pain [50] | Yes | Undefined | Yes | Yes | No | No | No | No | Yes | Consumers/ Caregivers | Yes |
| Spinal cord stimulation: Placement and management [51] | Yes | ESCS | No | Yes | No | No | No | No | No | Consumers/ Caregivers | Yes |
| Spinal cord stimulation [52] | Yes | ESCS | No | Yes | Yes | Yes | Yes | Yes | Yes | Both | Yes |
| Spinal Cord Stimulation's Role in Managing Chronic Disease PDF Symptoms [53] | Yes | ESCS | Yes | Yes | No | Yes | Yes | No | Yes | Consumers/ Caregivers | No |
| **For-profit (N=20)** |  |  |  |  |  |  |  |  |  |  |  |
| Spinal Cord Stimulator (SCS) Systems [54] | No | ESCS | Yes | Yes | No | No | No | No | Yes | Both | Yes |
| A NON-OPIOID ALTERNATIVE [55] | Yes | ESCS/ TSCS | No | Yes | No | Yes | Yes | No | Yes | Consumers/ Caregivers | Yes |
| SPINAL CORD STIMULATION [56] | Yes | Undefined | No | Yes | Yes | Yes | Yes | Yes | Yes | Both | Yes |
| Spinal Cord Stimulation (a) [57] | Yes | ESCS | No | Yes | No | Yes | Yes | Yes | Yes | Consumers/ Caregivers | Yes |
| Spinal Cord Stimulator Surgery – The risks and recovery time [58] | Yes | ESCS | No | Yes | Yes | Yes | No | Yes | Yes | Consumers/ Caregivers | Yes |
| Spinal Cord Stimulation (SCS) [59] | Yes | ESCS | No | Yes | No | Yes | No | Yes | Yes | Consumers/ Caregivers | Yes |
| Spinal Cord Stimulation Implant [60] | Yes | ESCS | No | Yes | No | No | No | Yes | Yes | Consumers/ Caregivers | Yes |
| THE NEED FOR SPINAL CORD STIMULATION [61] | Yes | ESCS | No | Yes | Yes | Yes | No | Yes | Yes | Consumers/ Caregivers | Yes |
| Spinal Cord Stimulator [62] | Yes | ESCS | Yes | Yes | Yes | Yes | No | Yes | Yes | Consumers/ Caregivers | Yes |
| Spinal Cord Stimulators: Procedure and Recovery [63] | Yes | ESCS | No | Yes | Yes | Yes | Yes | Yes | Yes | Consumers/ Caregivers | Yes |
| Spinal Cord Stimulator [64] | Yes | ESCS | No | Yes | No | Yes | Yes | Yes | Yes | Consumers/ Caregivers | Yes |
| SPINAL CORD STIMULATION SYSTEMS HEALTHCARE PROFESSIONALS [65] | No | ESCS | No | No | No | No | Yes | No | No | Both | No |
| What You Need to Know About Spinal Cord Stimulator Surgery [66] | Yes | ESCS | No | Yes | Yes | Yes | Yes | No | Yes | Consumers/ Caregivers | Yes |
| Spinal Cord Stimulator Review: Disadvantages And Risks Of The Surgery Implant [67] | Yes | ESCS | Yes | Yes | No | Yes | Yes | No | Yes | Both | Yes |
| Pain Management with Spinal Cord Stimulation Therapy [68] | Yes | ESCS | No | Yes | No | Yes | Yes | Yes | Yes | Consumers/ Caregivers | Yes |
| Spinal Cord Stimulation - Surgical Procedure [69] | Yes | ESCS | No | No | No | Yes | No | Yes | Yes | Consumers/ Caregivers | Yes |
| Spinal Cord Stimulator Trial / Implants [70] | Yes | ESCS | Yes | Yes | No | Yes | No | Yes | Yes | Consumers/ Caregivers | Yes |
| The Next Era of Personalization: WaveWriter Alpha™ SCS System [71] | No | ESCS | No | Yes | Yes | Yes | Yes | Yes | No | Clinicians/ Researchers | No |
| Types of Spinal Cord Stimulators: Functions, Differences, & Prices [72] | Yes | ESCS | Yes | Yes | No | No | Yes | No | Yes | Consumers/ Caregivers | Yes |
| Living With A Spinal Cord Stimulator: Answers To FAQs [73] | Yes | ESCS | Yes | Yes | Yes | Yes | Yes | Yes | Yes | Consumers/ Caregivers | Yes |
| **News/ Media (N=2)** |  |  |  |  |  |  |  |  |  |  |  |
| Spine Stimulator for Pain [74] | Yes | ESCS | No | Yes | No | No | No | Yes | Yes | Both | No |
| Spinal-cord stimulators help some patients, injure others [75] | Yes | ESCS | No | Yes | No | Yes | No | No | Yes | Both | No |
| **Personal/ blog (N=2)** |  |  |  |  |  |  |  |  |  |  |  |
| Anyone tried Spinal Cord Stimulation for Chronic Pain? [76] | No | ESCS | No | No | No | No | No | No | No | Consumers/ Caregivers | No |
| What is a Spinal Cord Stimulator? [77] | Yes | ESCS | No | Yes | Yes | Yes | Yes | No | No | Consumers/ Caregivers | No |
| Information Category: ^1^Definition or summary, ^2^SCS type/ subcategory, ^3^Indications, ^4^Contraindications, ^5^Side-effects or risks, ^6^Device considerations, ^7^Follow-up, ^8^Outcomes, ^9^Intended audience, ^10^Physician or clinic referral  †Both=Consumers/ Caregivers and Clinicians/ Researchers  Abbreviations: ESCS=Epidural Spinal Cord Stimulation, TSCS=Transcutaneous Spinal Cord Stimulation | | | | | | | | | | | |
